# A survived case of penetrating neck injury with intrathoracic organ damage

**DOI:** 10.1186/s40792-021-01163-1

**Published:** 2021-03-26

**Authors:** Atsushi Kagimoto, Takeshi Mimura, Nanami Hiraiwa, Yoshinori Yamashita

**Affiliations:** Department of General Thoracic Surgery, National Hospital Organization, Kure Medical Center and Chugoku Cancer Center, 3-1, Aoyama-cho, Kure, Hiroshima 737-0023 Japan

**Keywords:** Penetrating neck injury, Stab wound, Internal jugular vein injury, Intrathoracic organ damage

## Abstract

**Background:**

Thoracic surgeons rarely encounter stab wounds with injury to the intrathoracic organs. However, such sudden and urgent situations could arise; therefore, experiences in managing such cases are invaluable.

**Case presentation:**

An 84-year-old woman with depression who had a stab injury in the neck caused by a broad-bladed kitchen knife was brought to our facility by ambulance. She was stable in the emergency room; however, a computed tomography scan revealed that the blade had penetrated the right thoracic cavity. A right hemopneumothorax was seen. Considering the possibility of injury to the major vessels, a median sternotomy was performed. During the dissection around the blade, the patient started bleeding profusely, which required repair of an injury to the right internal jugular vein. The blade tip had penetrated the dorsal right upper lung lobe; however, it did not reach the hilum, and the knife was carefully removed. The damaged area of the lung was removed by wedge resection.

**Conclusion:**

Patients with deep stab wounds from knives are often hemodynamically stable because the blade acts as tamponade and prevents hemorrhage. Therefore, a surgical approach that allows for good visualization should be considered for the extraction of the blade.

**Supplementary Information:**

The online version contains supplementary material available at 10.1186/s40792-021-01163-1.

## Background

Thoracic surgeons rarely encounter stab wounds with injury to the intrathoracic organs. However, such sudden and urgent situations could arise; therefore, experiences in managing such cases are invaluable. We present a case of penetrating neck injury with damage to the intrathoracic organs and an impactful appearance that we treated successfully.

## Case presentation

**Fig. 1:**
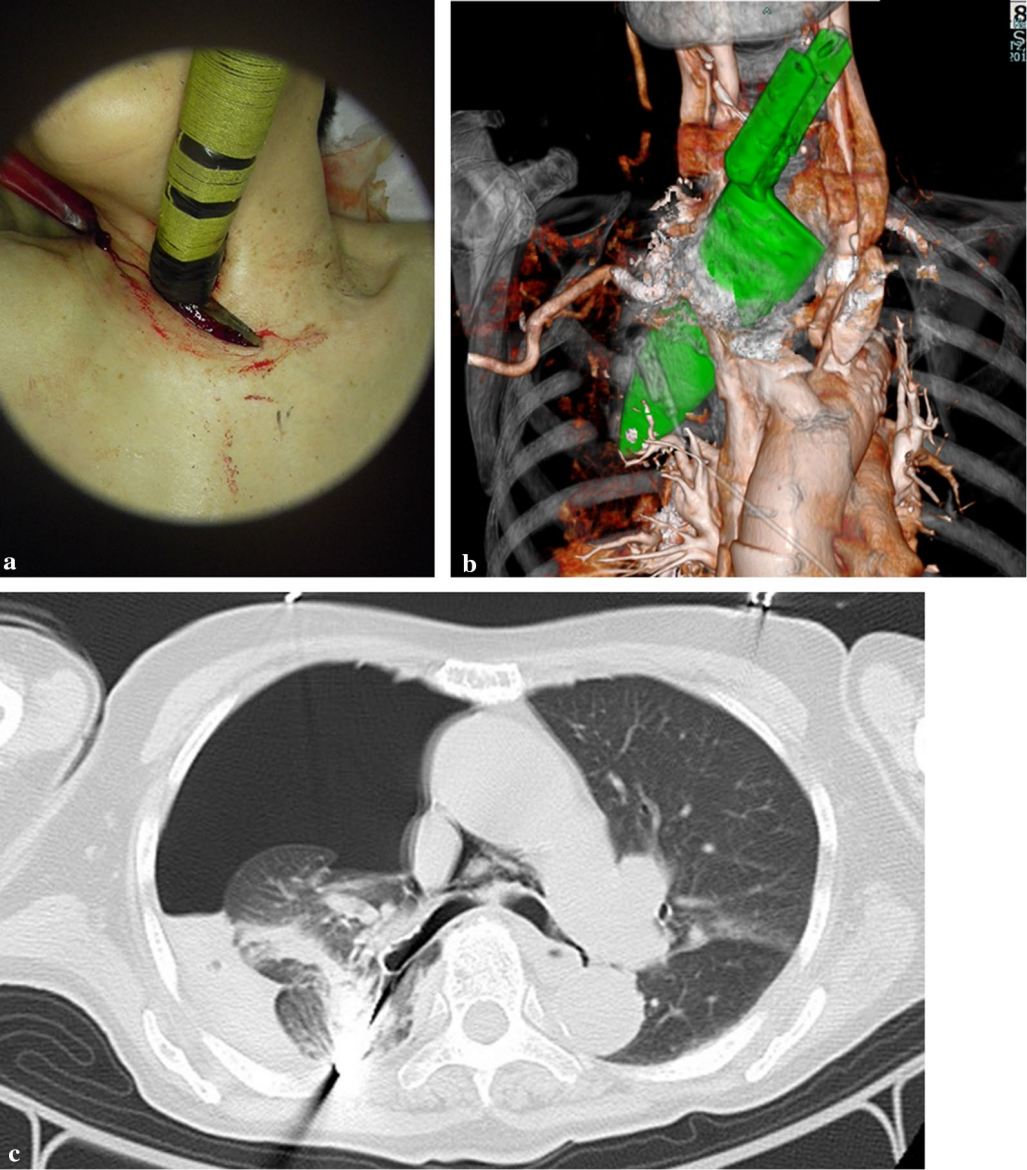
Preoperative image. **a** A broad-blade kitchen knife penetrating the neck. **b** Three-dimensional reconstruction from the preoperative computed tomography scan. The knife pierced through the left side of the right brachiocephalic vein and into the right thoracic cavity. **c** Chest computed tomography showed a right hemopneumothorax with collapse of about half of the right lung and a small amount of thoracic effusion

An 84-year-old woman with depression had a stab injury in the neck and was brought to our facility in an ambulance. There was a broad-bladed kitchen knife lodged above her substernal notch with about 15 cm of the blade inside her body (Fig. 1a). The patient was conscious and able to speak. Her blood pressure and heart rate were 187/126 mmHg and 127 bpm, respectively, and her percutaneous oxygen saturation was 98% under 5 L/min of oxygen administration. The computed tomography (CT) scan showed that the blade had penetrated the right thoracic cavity from the left side of the right brachiocephalic vein. A right hemopneumothorax, with collapse of about half of the right lung and a small amount of thoracic effusion, was observed, and laceration of the right upper lobe (RUL) was suspected. It was unclear at that time whether there were any massive vascular or hilar injuries (Fig. 1b, c). Therefore, considering the importance of securing the blood vessels, we decided to extract the knife in the operating room. A cardiovascular surgeon was included in the surgical team considering the possible need for extracorporeal membrane oxygenation and repair of large vascular injuries.

**Fig. 2:**
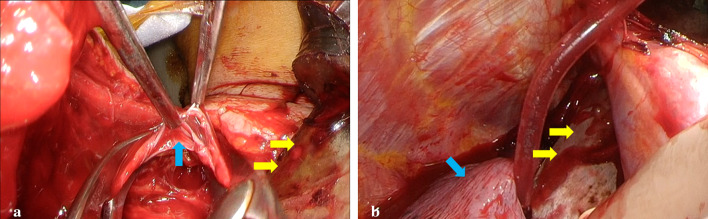
Intraoperative image. **a** The right internal jugular vein is damaged (blue arrow) by the knife (yellow arrow). **b** The knife (yellow arrow) penetrated between the right brachiocephalic artery and vein and reached the right pleural cavity. The blade tip had penetrated the dorsal right upper lobe (blue arrow)

After careful induction of anesthesia, a median sternotomy was performed, and the right pleural cavity was opened (Additional file 1: Video S1). The blade had penetrated between the right brachiocephalic artery and vein and had reached the right pleural cavity. While dissecting around the blade, the patient suddenly began to bleed, and hemostasis was achieved by compression with a cotton swab (Naruke-type Thoraco-cotton™, KENZMEDICO CO., LTD., Saitama, Japan). A 1.5-cm injury to the right internal jugular vein was found (Fig. 2a). The vascular injury was repaired by continuous sutures with 5-0 PROLENE® (Ethicon, Somerville, NJ, USA), clamping the proximal and distal sides. The blade tip was embedded in the RUL but did not reach the hilum; therefore, we were able to carefully extract the blade (Fig. 2b). The damaged RUL area was removed by wedge resection with automatic suturing instruments (Tri-Staple™ Radial Reload Black cartridge and Tri-Staple™ Black cartridge 60 mm with the Signia™ stapling system, Medtronic, Minneapolis, MN, USA). There was no injury to the vagus nerve or any other nerves. On postoperative day (POD) 3, the patient was weaned from mechanical ventilation, and the right chest drain was removed. On POD 5, she was transferred to a psychiatric ward for the treatment of depression. She has remained healthy for 3 years with no infections or other complications.

## Discussion

General thoracic surgeons, especially those in developed countries, rarely encounter patients with stab wounds that penetrate the thoracic organs. However, it is important to plan the treatment strategy in such cases as these injuries have high mortality [1]. In a South African study about the management of injuries with retained knife blades comprising 33 patients, simple extraction was performed in 19 patients (58%), and bleeding occurred only in one patient (5%) [2]. In a case series of 154 stab wounds associated with an event of terrorism in Israel, 4 patients developed hemodynamic collapse after extraction of the impaled knife [1]. Patients with retained blades and injuries to the major vessels might be hemodynamically stable on admission because the blade can act as tamponade and prevent hemorrhage. Therefore, in cases of a retained blade with a suspected vascular injury in the thoracic cavity, the blade should be extracted following a surgical approach that allows for adequate exposure and securing control of the proximal and distal vascular structures before removal of the impaled blade. In this case, the patient was conscious and able to speak in the emergency room. However, intraoperatively, there was sudden bleeding during dissection around the blade. If we had attempted a simple extraction before the surgery, we might not have been able to save her life. The incidence of complications, such as infection or neurological defects after surgery for stab wounds is high [1]; hence, careful management is important.

Neck injuries are divided into three distinct zones: 1, 2, and 3 [3]. Zone 1, which was the area applicable to this case, is defined inferiorly by the clavicle/sternal notch and superiorly by the horizontal plane that passes through the cricoid cartilage. Zone 1 injuries are less common, accounting for only 10% of neck injuries [4]; however, they have a high rate of fatality due to the presence of many important blood vessels in this region [5]. In addition, intrathoracic organ injuries often coexist with penetrating neck injuries [6]. Considering such possibilities, safer treatment options should be planned. It is essential to consult with a cardiovascular surgeon for assistance in preparing for the procedures involving injuries to the large vessels and the use of extracorporeal membrane oxygenation.

If the patient’s vital signs are stable, routine radiographic examinations are recommended for deep stab wounds to define the relationships between the implements and major visceral structures and vessels [6]. In our case, a CT scan was performed; however, the vascular injury could not be completely visualized due to the presence of an artifact. Angiography is useful and recommended when the presence or absence of vascular injury is obscured by artifacts, especially if the patient's vital signs are stable [7, 8]. However, in our case, the broad-bladed kitchen knife was still entrenched in the patient’s neck, and it was important to remove the knife as soon as possible to prevent a state of shock, which could have occurred even with a slight movement by the patient. The outcome in this case was good, due to immediate surgery with median sternotomy, considering the repairs of major vessels or lung resection. This approach provided a good visual field and prevented a potential catastrophe. If the median sternotomy would have failed to provide a good visual field, we would have made an additional incision, such as a high anterolateral thoracotomy or supraclavicular incision. In patients with deep thoracic stab wounds, the surgical approach should be selected considering all the possible situations.

## Conclusion

We successfully treated a case of penetrating neck injury with intrathoracic organ damage. When treating patients with deep stab wounds caused by knives, major vessel or visceral organ damage should be suspected, and the retained blade should be extracted by a surgical approach that allows for good visualization.

## Supplementary Information

**Additional file 1:** Intraoperative findings of extraction of the embedded knife.

## Data Availability

Not applicable.

## Abbreviations

CT: Computed tomography
RUL: Right upper lobe (RUL)
POD: Postoperative day (POD)
